# A multiple criteria decision analysis to establish the use cases and candidate point of care tests to enter into a platform trial of multiple *in vitro* diagnostic point of care tests in the prehospital environment

**DOI:** 10.3310/nihropenres.13580.1

**Published:** 2024-05-09

**Authors:** Kim Kirby, Jessica Coggins, Andy Gibson, Cathy Liddiard, Theresa H.M. Moore, Jelena Savović, Kimberley Mitchell, Alexander Thompson, Jonathan Benger, Richard Body

**Affiliations:** 1 University of the West of England, Bristol, BS16 ODD, UK; 2 South Western Ambulance Service NHS Foundation Trust, Exeter, EX2 7HY, UK; 3 The National Institute for Health and Care Research Applied Research Collaboration West, Bristol, BS1 2NT, UK; 4 Population Health Sciences, Bristol Medical School, University of Bristol, Bristol, England, UK; 5 University of Manchester, Manchester, M13 9PL, UK

**Keywords:** Multiple Criteria Decision Analysis, Emergency Medical Services, Point of Care Testing

## Abstract

**Background:**

There are increasing demands on Emergency Medical Services. More efficient treatment pathways are required to support conveyance decision making and patient referral in prehospital care. Point of Care testing is increasingly available and utilised across the NHS to support optimal ways of working. We aimed to design and conduct a Multiple Criteria Decision Analysis to prioritise in vitro point of care tests and use cases for inclusion in a platform trial of in vitro point of care testing in UK Emergency Medical Services.

**Methods:**

We designed a Multiple Criteria Decision Analysis that included systematic scoping reviews stakeholder recruitment, two stakeholder surveys and two stakeholder workshops to scope the use cases, explore criteria and map use cases, evaluate the criteria and measure the use cases against the criteria.

**Results:**

We recruited 32 stakeholders. We developed a scoring matrix with 4 criteria for scoring the use cases and 8 criteria for scoring the point of care tests and applied weighting determined from survey results. Use cases were scored by the stakeholders against 4 criteria. The 3 highest scoring use cases were point of care troponin testing in: possible Acute Myocardial Infarction, lactate testing in suspected sepsis and in trauma. We developed the process for scoring the point of care tests to be completed close to a proposed trial to allow for a changes in technology.

**Conclusions:**

We successfully designed a Multiple Criteria Decision Analysis to identify use cases and candidate tests for inclusion in a future platform trial of in vitro point of care testing in UK Emergency Medical Services. We identified 3 use cases for evaluation in a platform trial of in vitro point of care testing: troponin testing in possible acute myocardial infarction, lactate testing in suspected sepsis and lactate testing to identify occult haemorrhage in trauma.

## Plain Language Summary

**Aim of the research:**

To identify the most useful bedside tests and patient conditions appropriate for bedside testing in ambulance services.

**Background to the research:**

Tests called point of care tests can be conducted at a patient’s side and quickly give clinicians information that can help with decision-making about the right treatment and pathway for patients. There are many of these tests available and we wanted to complete a trial to understand if tests are suitable for use in ambulance services and are the right tests for patients to have. Before we could conduct a trial we needed to understand which patient conditions are amenable to point of care testing in ambulance services and which tests are appropriate to use in this environment.

We conducted research to design and complete a Multiple Criteria Decision Analysis (MCDA). MCDA is a method that allowed us to identify the most appropriate tests and conditions to enter into a trial of point of care testing in ambulance services.

**Design and methods used:**

We reviewed the relevant literature to understand which tests were available and which conditions were appropriate for point of care testing in ambulance services. We completed surveys and workshops with stakeholders to identify criteria and a scoring system to score the tests and conditions that those tests can be used.

**Results:**

We scored the conditions in which point of care tests could be used in ambulance services. We identified that point of care tests were most suitable for patients that might be suffering a heart attack, patients that have suffered a severe injury and patients that may have a severe infection.

## Introduction

Demand on Emergency Medical (Ambulance) Services (EMS) continues to increase ^1^. Lengthy delays in patient handover at crowded Emergency Departments (EDs) compromises the ability of EMS to respond to new emergency calls, leading to prolonged ambulance response times ^2^. Strategies to improve efficiency by maximising the safe avoidance of patient conveyance to hospital wherever possible, while also ensuring patients who are transported receive the best care and reach the most appropriate facility, are paramount.

Laboratory testing guides 60–70% of clinical decisions, and Emergency Departments utilise in vitro diagnostics for swift identification of serious conditions ^3^. In vitro Point of Care (POC) testing could similarly enhance EMS by achieving shorter response times, less hospital transfers and reduced ED waits, with potential for substantial cost savings. POC testing is increasingly available and utilised across the NHS. Rapid growth in the POC testing market is predicted and may rival the pharmaceutical industry within 20 years ^4^.

Platform trials are designed to test multiple interventions at the same time ^5^ and in 2023 the UK National Institute of Health and Care Research (NIHR) encouraged funding applications for its Health Technology Assessment (HTA) programme for platform trials of strategic priority ^6^. While planning a platform randomised evaluation of clinical and cost effectiveness of in vitro POC testing in EMS in the UK, we required a robust and transparent process to triage POC tests and associated “use cases” for inclusion in a platform trial. Multiple criteria decision analysis (MCDA) is a commonly used method to guide complex decision making in HTA ^7^ when there are multiple options and multiple relevant criteria to be taken into account during the decision-making process ^8^. In particular, MCDA has been most frequently used within HTA to guide resource priority decision-making ^9^. This paper describes the process and outcomes of MCDA methodology in the prioritisation of candidate in vitro POC tests and use cases for potential inclusion in a proposed future platform randomised controlled trial of in vitro POC testing in UK EMS. In this paper the term POC tests refers to in vitro POC testing.

## Methods

### Patient and Public Involvement

This work benefitted from a PPI co-applicant who had prior experience of relevant high-quality point of care testing research and who was involved in all aspects of the research. A wider group of Public Advisory Group members were involved in the following ways:

Public Contributors (PCs) helped us to understand the range of variation in service provision and patient circumstances. This included looking at the potential alternative care pathways that can be accessed by testing and describing the facilitators and barriers to pre-hospital testing from a patient/carer perspective. They also advised on the pre-requisites that make pre-hospital testing and alternative care pathways acceptable from a patient and carer perspective.

PCs were involved in the development of the Stakeholder Survey 1 to create the longlist of ‘criteria’ for assessment and the Stakeholder Survey 2 to define the weighting of criteria. PCs also participated fully in both stakeholder workshops.

We conducted an MCDA to prioritise candidate in vitro POC tests and use cases for inclusion in a platform trial of POC testing in UK ambulance services. The MCDA was conducted in accordance with the principles described in the good practice guidelines for the conduct of MCDA published by the International Society for Health Economics and Outcomes Research (ISPOR) ^10^.

**Figure 1.**
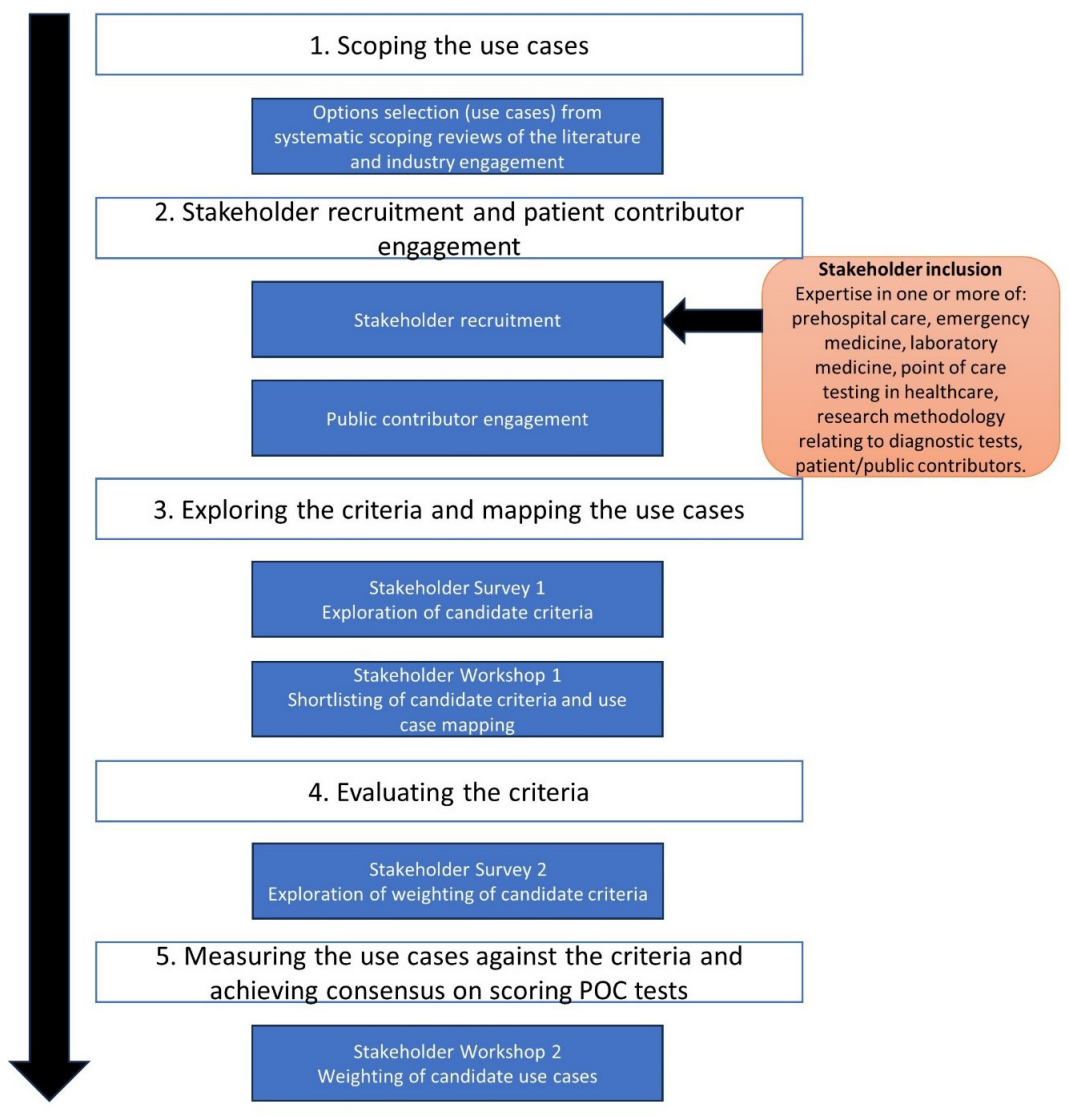
Multiple Criteria Decision Analysis flow diagram.

The MCDA was conducted between March and September 2023 and the process is illustrated in Figure 1.

### Scoping the use cases

***Identification of potential use cases***

The options (use cases) were identified through systematic literature search and selection process following principles of scoping review ^11^. We did not assess the bias in the studies identified, nor did we attempt any formal synthesis of the evidence as these are outside of the methods used for scoping reviews ^12^.

***Searching for relevant studies***

To identify POC tests used within, or with potential for use in the EMS we searched three electronic bibliographic databases (Medline and EMBASE on Ovid and CINAHL on Ebscohost). The search was developed by an information specialist (SD) and systematic reviewers (TM,JS) in liaison with the rest of the team. The strategy combined terms for point of care tests with terms for EMS. We did not apply a filter to restrict types of study design. The search strategies are available in an online data repository ^13^. We searched reference lists of key systematic reviews. We restricted to English language and research published after 2000 and focused the search on adults. Following an initial broad search for POC tests in the EMS, additional supplementary searches for specific potential use cases were completed between April and August 2023. A table summarising the searches is presented in Extended Data File 1. Titles and abstracts of records were screened by one author (TM) with all excluded records checked for exclusion by a second member of the wider team (see acknowledgements). Full-text versions of the records marked “retrieve” by either reviewer were screened by one author (TM) with all excluded papers checked for exclusion by a second member of the wider team. Strict inclusion and exclusion criteria were used to guide selection, criteria included: reporting on a POC test used in the EMS, setting was the EMS, POC test was using bodily fluids (saliva, blood, serum, breath, sweat). We excluded studies where the focus was use of equipment to obtain a diagnosis from a body scan, such as ultrasound. We excluded tests used in specialist services such as mobile stroke units because of the reliance on specialist personnel and scanning equipment in conjunction with any POC tests. Search data were managed using Rayyan and Endnote software ^14, 15^. Screening was managed using the Microsoft software Access and Excel ^16, 17^.

***Preparation of stakeholder summaries***

Data extraction from studies identified in the scoping exercise was done by one researcher (TM) and checked by a second (KM,RP). Data extracted included: Type of test(s), test device name and manufacturer, study design, condition the test was being used for, biomarkers being tested. Information for potential use cases was organised into summaries for stakeholders into 6 types of information, 1) systematic review of primary research, 2) Economic evaluation of use of POC tests in the EMS, 3) ability to make causal inference for a change in patient pathway, from a) experimental studies with interventions assigned at random, or b) from non-randomised studies with a comparator group or controlled before and after studies 4) demonstration of the POC test being used in the EMS from observational studies describing the use of a POC test(single arm observation studies) 5) Views of paramedics or clinicians or patients or hospital staff on the use of POC tests in the EMS to change the patient pathway (qualitative studies or surveys). Studies investigating the diagnostic test accuracy of POC tests were documented and listed but not summarised as this information is not sufficient to ascertain if a change in patient pathway has potential. The number of records for each use case, and the type of information identified for each were organised into tables and stakeholder summaries that were provided at two workshops. The summaries presented at Workshop 2 are listed in (Extended Data File 1).

### Stakeholder recruitment and patient contributor engagement

***Stakeholder recruitment***

The MCDA focused on UK EMS care, but recruited national and international stakeholders with the expertise to meet the inclusion criteria described in Figure 1. Stakeholders were identified and recruited during June and July 2023. Eligible stakeholders, who had been identified as lead authors of research found during the scoping review, or whose work and expertise was familiar to the study team, were contacted. Snowball sampling was then employed to recruit additional stakeholders. Initial contact was via an email providing information about the study. Where stakeholders expressed an interest in participating, they were provided with a participant information sheet and a consent form and asked to consent to participate in the study. In addition to seeking topic experts, we also recruited a sample of patient and public stakeholders from an established patient and public research advisory group.

***Public contributor workshops***

To support the full engagement of public contributors in the MCDA process we convened two virtual public contributor workshops. The initial workshop was designed to provide contributors with training on background information about the topic, the proposed platform trial, and the MCDA methodology. A member of the study team (KK) and an expert in Patient and Public Engagement (AG) presented information to the group and answered questions. Participants were also provided with written information. A subsequent workshop was convened and supported by JC, HN and AG to inform public contributors about the process that would be used in Workshop 2, and to ensure there was adequate support to enable patient and public contributors to engage fully in all activities.

### Exploring the criteria and mapping the use cases

***Stakeholder Survey 1***

Stakeholder Survey 1 (Extended Data File 2) was disseminated to non-public contributor stakeholders to explore candidate criteria that should be considered when deciding whether a POC test is suitable for use in EMS. The survey contained questions focused on ‘a priori’ themes of promise, plausibility, risk, costs, the nature of the condition being investigated, the technology, the adopter system, the wider institutional and societal context, the evidence quality and other criteria which we selected from reviews of pertinent research by Campbel and Knox ^18^ and Greenhalgh and colleagues ^19^. Stakeholders were asked to rate the overall importance of each of the criteria using a five-point Likert scale but were also asked to provide free text responses to elaborate upon their answers.

Responses to the survey were collated and reviewed by the research team. Likert scale responses were summarised using frequencies. Survey 1 results informed an update of a priori themes, producing a new shortlist of criteria to take forward into Stakeholder Workshop 1.

***Stakeholder Workshop 1***

Prior to the workshop summaries of the evidence available for different use cases were provided to the stakeholders. Responses to Survey 1 were visually summarised using an online interface (Miro board ^20^) and formed the basis of a subsequent discussion. During the online workshop, stakeholders were invited to map clinical pathways for candidate use cases (mapping both the current care pathway and the proposed amendments to the care pathway that introduction of a POC test would allow), discuss the importance of each shortlisted criterion and make suggestions or alterations to the shortlist. All ideas were captured on the Miro board and displayed to the stakeholders, who were asked to agree on the final content of the Miro board by the end of the workshop. The workshop was recorded using Microsoft Teams.

We then compared the refined shortlist of criteria against the Evidence and Value: Impact on Decision Making (EVIDEM) framework ^21^, which has previously been used for MCDA involving health technologies ^22^. Following an iterative process of coding and refinement within the study team, we reached a final consensus that the list of criteria identified was truly representative of stakeholder opinions, while being sufficiently specific and measurable to use in the MCDA process. The study team also prepared a draft rating system with which to score each criterion. We searched for published examples of established scoring systems and used these wherever possible. Where that was not possible, the team agreed three- or four-point scoring systems to put to the stakeholders.

### Evaluating the criteria

***Stakeholder Survey 2***

To validate and finalise the list of criteria to be used for scoring and to determine the weightings that would be used to score those criteria, we disseminated a second survey (Extended Data File 2) to non-public contributor stakeholders. Each of the shortlisted criteria was presented back to the stakeholders. They were asked three questions about each criterion. First, they were asked to score the importance of the criterion on an 11-point scale. This would later be used to assign weightings to the final criteria. Second, they were asked to provide feedback on the proposed rating system. They were asked if they felt that the rating system was appropriate. If not, we asked stakeholders to explain why not and to propose alternatives. Finally, we asked the stakeholders if any of the categories should be used to exclude a POC test from being considered in a future platform trial. This allowed us to identify the ‘deal breakers’ or satisfice criteria, which would exclude a POC test from further consideration.

The responses to the survey were collated and analysed within the study team. The list of criteria was finalised and stratified into two sections: criteria for prioritising use cases; and criteria for prioritising individual POC tests or assays within those use cases. Scoring systems and satisfice criteria were finalised based on consensus of the stakeholders, with review within the study team for every instance of disagreement. Outputs were taken forward into Stakeholder Workshop 2.

### Measuring the use cases against the criteria and achieving consensus on scoring POC tests

***Stakeholder Workshop 2***

Both expert and public contributor stakeholders were invited to participate in the online Stakeholder Workshop 2. Prior to the workshop, all stakeholders were provided with the final list of options (use cases) identified alongside their associated care pathways (current and POC test-driven), which had been mapped earlier in the project (Extended Data File 4). A summary of the evidence identified in the updated systematic scoping exercise was also provided for each use case (Extended Data File 1).

During the first half of the second workshop, the final list of criteria for scoring use cases was presented back to all stakeholders, who were given an opportunity to make any final adjustments. Once consensus had been achieved, we proceeded to the second stage of the workshop, during which the options (candidate use cases) were presented and scored. The relevant care pathways were explained to stakeholders, and further comment was invited. The summary of evidence for each use case was then presented. The summary was provided in language that was comprehensible to a lay person, and there was an opportunity for all stakeholders to ask questions.

Once we had confirmed that the stakeholders had understood the information and after answering any questions, we proceeded to invite the stakeholders to score the options against each of the agreed criteria using the scoring systems developed earlier in the project. This was done via Mentimeter ^23^. Average scores from Survey 2 provided the weighting figure assigned to each criteria. The pre-determined weightings and satisfice criteria were used to create a template scoring matrix prior to the workshop. Once stakeholders had provided a score, this was averaged (mean) and added to the matrix by a study team member during the workshop. The weighting and average figures were multiplied for each criteria, and this provided a final score for each use case.

By collating the scores contemporaneously, we were then able to present the final results back to the stakeholders and ensure that consensus had been achieved about the final scores and the ranking of use cases. Having ranked the use cases, we selected the top three scoring use cases for inclusion in a future platform trial.

***Achieving consensus on scoring POC tests***

Prior to completing Stakeholder Workshop 2, we also presented the agreed criteria for scoring in vitro POC tests and scoring systems for rating individual tests/assays within the relevant use cases. Consensus was achieved for each prior to the end of the workshop. We then identified all eligible commercially supplied, CE- or UKCA-marked assays for the relevant test. This list will be updated and POC tests scored prior to a platform trial of in vitro POC tests in EMS to allow for industry advancements and for the study team to systematically review each device before scoring.

### Ethics

Ethics committee approval was granted by the University of the West of England, Bristol Faculty Research Ethics Committee reference: UWE REC REF No: HAS.23.04.101.

## Results

### Scoping the use cases

***Options (use case) - Identification***

Systematic scoping reviews of POC tests in EMS supplemented by surveys of industry organisations, identified the candidate use cases shown in Table 1.

**Table 1.** Use cases and POC tests identified during systematic literature reviews.

Use Case
Lactate testing in trauma
SARS-CoV-2 testing in suspected respiratory tract infection
Lactate testing in sepsis
Troponin for acute myocardial infarction
Coagulation testing in trauma
Blood gas testing in COPD
Blood ketone testing to diagnose diabetic ketoacidosis
NT-proBNP to identify heart failure (natriuretic peptides)
NT-proBNP to identify sepsis
C-reactive protein (CRP) testing in sepsis
Beta-HCG testing to identify pregnancy (using whole blood)
Biomarkers of traumatic brain injury

### Stakeholder recruitment and patient contributor engagement

***Stakeholder recruitment***

We recruited 32 stakeholders to participate in the study and their expertise and country of origin is shown in Table 2.

**Table 2.** Stakeholder expertise and country of employment.

Number	Area of expertise	Country
1	Evaluating complex interventions in prehospital care	UK
2	Prehospital care, point of care testing, prehospital research	UK
3	Point of care testing in healthcare	UK
4	Point of care testing in healthcare	UK
5	Point of care testing in healthcare	UK
6	Point of care testing in healthcare	UK
7	Prehospital care, Prehospital research	UK
8	Point of care testing in healthcare	UK
9	Point of care testing in healthcare	New Zealand
10	Point of care testing in healthcare	UK
11	Laboratory medicine, point of care testing in healthcare	Norway
12	Prehospital care	UK
13	Point of care testing in healthcare	UK
14	Prehospital care	UK
15	Prehospital care	UK
16	Prehospital care, point of care testing healthcare	UK
17	Prehospital care	UK
18	Prehospital care, point of care testing in healthcare	Netherlands
19	Prehospital care, point of care testing in healthcare	US
20	Prehospital care, point of care testing healthcare	UK
21	Point of care testing in healthcare	UK
22	Research methodology relating to diagnostic tests	UK
23	Research methodology relating to diagnostic tests	UK
24	Prehospital care	UK
25	Patient/public contributor	UK
26	Patient/public contributor	UK
27	Patient/public contributor	UK
28	Patient/public contributor	UK
29	Prehospital care	UK
30	Prehospital care, prehospital research, point of care testing healthcare	UK
31	Prehospital care	UK
32	Prehospital care, point of care testing healthcare	UK

### Exploring the criteria and mapping the use cases

***Stakeholder Survey 1***

Fifteen “expert” participants (54% response rate) completed Stakeholder Survey 1 in May and June 2023.

**Figure 2.**
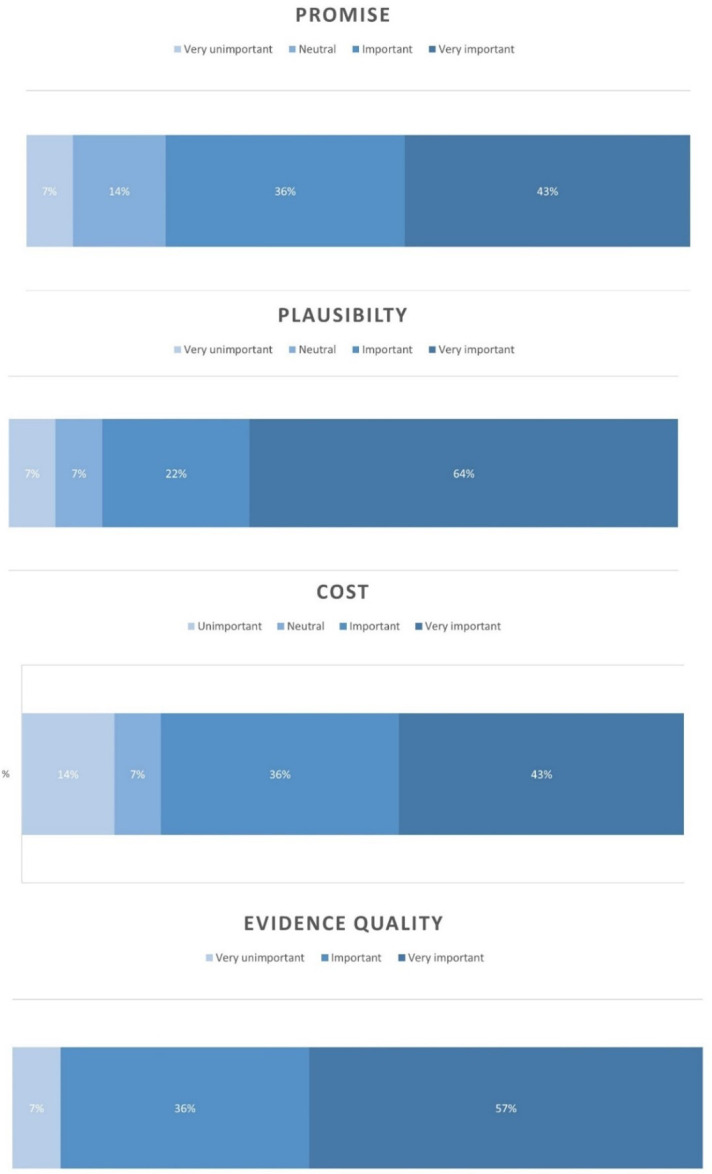
Survey 1 charts.

Charts displaying the results from the Likert questions are shown in Figure 2. Free text responses to Survey 1 are included in Extended Data File 3.

***Stakeholder Workshop 1***

Stakeholder Workshop 1 was conducted on June 7 ^th^ 2023 with 13 stakeholder participants. The findings of Survey 1 were discussed during the workshop and a list of criteria to continue into Survey 2 for scoring and weighting was discussed and developed. These are shown in Table 3.

**Table 3.** List of candidate criteria developed from the findings of survey 1.

List of criteria
Potential for the POC test to alter the clinical pathway
Size of the population affected
Suitability for use in the prehospital environment
Suitability of the weight of the POC test for prehospital use
Suitability of the turnaround (test completion) time of the POC test for prehospital use
Evidence of diagnostic accuracy
Evidence of the clinical effectiveness of the POC test
Risk of the POC test
Sustainability of the manufacturing process (green impact)
Integration with ambulance systems
Precision (reproducibility and reliability of the test method) of the POC test

Use cases were mapped and discussed in Stakeholder Workshop 1 and through consensus the most appropriate use cases for further analysis were identified, based principally on available technology and pathway readiness. Identified use cases were ketone testing in diabetic ketoacidosis, NT-proBNP testing in heart failure, lactate testing in trauma, lactate testing in sepsis, bacterial or viral testing in respiratory tract infection, biomarker testing in traumatic head injury and troponin testing in chest pain. Use case mapping is included in Extended Data File 4.

### Evaluating the criteria

***Stakeholder Survey 2***

Fifteen expert participants (54% response rate) completed Survey 2 in August 2023. The results are included in Extended Data File 6. Table 4 details the scoring and satisfice results of Survey 2.

**Table 4.** Scoring and satisfice results Survey 2.

	Average	Median	Minimum	Maximum	Satisfice criteria
**Criteria for prioritising use cases**					
Importance of potential impact on the patient’s care pathway	7.2	7	3	10	None
Importance of size of the population affected	5.9	6	2	9	None
Importance of evidence of diagnostic accuracy of POC tests in this use case	8.8	9	6	10	0,1,2
Importance of the evidence of the clinical effectiveness of the POC Test in this use case	7	8	2	10	4
**Criteria for prioritising POC tests**					
Importance of suitability of the POC test power supply for prehospital use	8	8	5	10	0
Importance of suitability of the size of the POC Test for prehospital use	7	8	5	10	0
Importance of suitability of the weight of the POC Test for prehospital use	6	7	0	8	0
Importance of suitability of the turnaround (test completion) time of the POC Test for prehospital use	8	8	5	9	0
Importance of risk of the POC Test	8	8	5	9	0
Importance of the sustainability of the manufacturing process for the POC Test	5	5	1	8	0
Importance of the integration of the POC Test with ambulance systems	7	6	1	10	0
Importance of the precision (reproducibility and reliability of the test method) of the POC Test	9	9	7	10	To be further developed

Options meeting satisfice criteria (coagulation testing in trauma, blood gas testing in COPD, NT-proBNP in heart failure and sepsis, C-reactive protein in sepsis, Beta-HCG in pregnancy, biomarkers of traumatic brain injury) were automatically excluded from entering the platform trial because they met the satisfice criteria. This left five use cases to be scored during Stakeholder Workshop 2.

### Measuring the use cases against the criteria and achieving consensus on scoring POC tests

***Stakeholder Workshop 2***

There were 14 participants in Stakeholder Workshop 2. Participants used Mentimeter ^23^ to score each of the criteria for the five included use cases, this is detailed further in Table 5. Weighting was applied to the criteria: Potential to alter the clinical pathway; Certainty of evidence for diagnostic accuracy; Certainty of evidence for clinical effectiveness; Size of population affected. The weighting applied was the average ‘importance’ scoring given to the criteria by participants in Survey 2. The full Mentimeter ^23^ results are included in Extended Data File 7 and Table 6 illustrates the results of the use case scoring.

**Table 5.** Scoring of use case criteria.

Criteria	Categories
Potential to alter the clinical pathway	0 No/Very low potential 1 Low potential 2 Moderate potential 3 High potential
Classing of certainty of evidence for diagnostic accuracy	0 Absent 1 Very low 2 Low 3 Moderate 4 High
Certainty of evidence for clinical effectiveness	0 Absent 1 Very low 2 Low 3 Moderate 4 High
Size of the population affected	Call Volume 0 <1% 1 2.9% 3 4.9% 4 >=5%

**Table 6.** Results of scoring use cases.

Criterion	Use cases:	*Troponin AMI*	*Natriuretic peptides AHF*	*Lactate sepsis*	*Lactate trauma*	*Ketones for DKA*
***Potential to improve the care pathway***	Weighting assigned to the criterion	7.20				
	Average score given by the stakeholders	2.85	2.42	2.54	2.15	2.54
	Satisfice criteria met?	No	No	No	No	No
	Final score	**27.32**	**23.19**	**24.36**	**20.67**	**24.36**
***Certainty of evidence for diagnostic accuracy***	Weighting assigned to the criterion	8.8				
	Average score given by the stakeholders	3.33	2.15	3.08	2.62	1.69
	Satisfice criteria met?	No	No	No	No	Yes
	Final score	**29.33**	**18.95**	**27.08**	**23.02**	**14.89**
***Certainty of evidence for clinical effectiveness***	Weighting assigned to the criterion	7.0				
	Average score given by the stakeholders	3.17	0.42	2.15	1.62	1.77
	Satisfice criteria met?	No	No	No	No	No
	Final score	**22.17**	**2.92**	**15.08**	**11.31**	**12.38**
***Size of population affected***	Weighting assigned to the criterion	5.90				
	Average score given by the stakeholders	0	0	1	1	0
	Satisfice criteria met?	No	No	No	No	No
	Final score	**0.00**	**0.00**	**5.90**	**5.90**	**0.00**
	**Total**	**78.82**	**45.06**	**72.42**	**60.89**	**51.64**

The three highest scoring use cases; POC troponin testing in possible Acute Myocardial Infarction (AMI), POC lactate testing in suspected sepsis and POC lactate testing in trauma, were prioritised for evaluation within a future platform trial of POC testing in EMS.

***Achieving consensus on scoring POC tests***

The satisfice criteria for evaluating the POC tests detailed was agreed with the stakeholders during Stakeholder Workshop 2 and this is detailed within Table 4.

## Discussion

We developed an MCDA process for identifying the most appropriate options (use cases and in vitro POC tests) to enter into a platform trial of POC testing in UK EMS. The process consisted of identifying relevant use cases and POC tests through systematic searching of the literature and industry engagement. We identified a relevant stakeholder group and worked with these stakeholders to: scope the use cases; explore the criteria and map the use cases; evaluate the criteria; measure the use cases against the criteria and achieve consensus on scoring in vitro POC tests. Following this process, we identified that the most appropriate use cases to progress into a subsequent platform trial are POC troponin testing in possible AMI, POC lactate testing in suspected sepsis and POC lactate testing in trauma.

There is a general recognition that there are few resources to support organisations in evaluating POC tests for implementation, and that often POC tests are developed that appear to meet clinical requirements, but then fail to be adopted successfully into practice ^24^. There are no studies that investigate an MCDA in relation to POC testing specifically. This study has developed a formal and reproducible methodology to evaluate alternatives and priorities for use cases and POC tests that may be included in a platform trial of POC testing in UK EMS, facilitating transparency in decision-making. Our study presents a quantitative MCDA as opposed to qualitative, or MCDA with decision rules ^10^. Quantitative MCDA is considered more robust because the use of scores and weightings improves the consistency of recommendations.

There are well-documented benefits of using MCDA for HTA. These include a sound structuring of the decision problem, avoidance of bias in complex decision-making, and a transparent, consistent and reproducible process ^25^. The method is particularly attractive when there are many important criteria to consider when triaging the available options. These qualities are important in the prioritisation of POC tests to enter into a platform trial of POC testing in UK EMS. In addition, our process of MCDA has included a wide range of stakeholders, including public contributors allowing greater participation in decision-making. An argument against using MCDA in HTA, and a factor that we recognise as a potential limitation in our methods, is that the management of the scoring and weighting of factors by researchers can inhibit debate, rather than stimulate it ^25^. However, our stakeholders were informed of all decision-making and indicated throughout the process that they were content with the methods used and the outcomes. Other limitations include our approach to the selection of stakeholders, which relied on volunteers who may not be wholly representative of the wider community, and the requirement to use relatively subjective assessments in some areas of the MCDA. It is possible that a different group of stakeholders may have reached somewhat different conclusions.

Moving forward we will further refine and develop this MCDA process and continue to utilise it to determine which POC tests are appropriate for inclusion in an EMS platform trial. This research will also be informative for healthcare organisations and guideline decision makers on the appropriateness of POC tests for EMS and related settings, recognising that this MCDA was completed with UK EMS in mind and may not be generalisable to international settings. Continuation of research in this area in a future trial of POC testing in EMS is warranted to understand the factors that influence successful adoption and implementation of POC testing in UK EMS.

## Conclusion

We report on the development and use of MCDA methodology to identify candidate tests for inclusion in a future platform trial of POC testing in UK EMS. After identifying stakeholders and candidate tests (options) through systematic scoping reviews and surveys of industry organisations, we used a series of stakeholder surveys and workshops to define the criteria for selection of use cases and in vitro POC tests, and determine the appropriate weighting and scoring criteria. Through this process, we identified three use cases for evaluation in a platform trial: troponin testing in possible AMI, lactate testing in suspected sepsis and lactate testing to identify occult haemorrhage in trauma.

## Ethics and consent

Ethics committee approval was granted on 2 ^nd^ June 2023 by the University of the West of England, Faculty of Health and Applied Sciences Faculty Research Ethics Committee, reference: UWE REC REF No: HAS.23.04.101. All participants in this research provided written informed consent prior to their participation.

## Data Availability

Open Science Framework: PrePOCTED Multiple Criteria Decision Analysis Extended Data. https://doi.org/10.17605/OSF.IO/3YUBA ^26^. This project contains the following underlying data: Extended Data file 1. (Search plan for point of care tests in the emergency medical services, and evidence summaries presented to stakeholder workshops) Extended Data file 2. (Stakeholder Survey 1) Extended Data file 3. (Stakeholder Survey 1 results) Extended Data file 4. (Clinical Pathway Mapping) Extended Data file 5. (Stakeholder Survey 2) Extended Data file 6. (Stakeholder Survey 2 Results) Extended Data file 7. (Workshop 2 Meni-Meter results) Data are available under the terms of the Creative Commons Zero "No rights reserved" data waiver (CC0 1.0 Public domain dedication).
