# Supplementary material for: A multiple criteria decision analysis to establish the use cases and candidate point of care tests to enter into a platform trial of multiple in vitro diagnostic point of care tests in the prehospital environment
Source: NIHR Open Res. 2024 Jul 23;4:29. Originally published 2024 May 9. [Version 2] doi: 10.3310/nihropenres.13580.2 (PMC11320030; doi:10.3310/nihropenres.13580.2)
Supplement: Peer review history

# Reviewer report: Reviewer response for version 2

**DOI:** 10.3310/nihropenres.14854.r32407

**Published:** 2024-08-22

**Authors:** Rudolf T Tolsma

**Affiliations:** 1 Emergency Medical Service, Ambulance IJsselland, Zwolle, The Netherlands

No new comments

Is the work clearly and accurately presented and does it cite the current literature?

Yes

If applicable, is the statistical analysis and its interpretation appropriate?

Not applicable

Are all the source data underlying the results available to ensure full reproducibility?

Yes

Is the study design appropriate and is the work technically sound?

Yes

Are the conclusions drawn adequately supported by the results?

Yes

Are sufficient details of methods and analysis provided to allow replication by others?

Yes

Reviewer Expertise:

Prehospital care, riskstratification, point of care testing in healthcare

I confirm that I have read this submission and believe that I have an appropriate level of expertise to confirm that it is of an acceptable scientific standard.

# Reviewer report: Reviewer response for version 2

**DOI:** 10.3310/nihropenres.14854.r32406

**Published:** 2024-08-07

**Authors:** Daniel Ebbs

**Affiliations:** 1 Yale University, New Haven, Connecticut, USA

Authors addressed concerns adequately for full approval for indexing.

Is the work clearly and accurately presented and does it cite the current literature?

Yes

If applicable, is the statistical analysis and its interpretation appropriate?

Partly

Are all the source data underlying the results available to ensure full reproducibility?

Yes

Is the study design appropriate and is the work technically sound?

Yes

Are the conclusions drawn adequately supported by the results?

Partly

Are sufficient details of methods and analysis provided to allow replication by others?

Partly

Reviewer Expertise:

Prehospital emergency services, Prehospital POCTs, Prehospital Predictive Modeling

I confirm that I have read this submission and believe that I have an appropriate level of expertise to confirm that it is of an acceptable scientific standard.

# Reviewer report: Reviewer response for version 1

**DOI:** 10.3310/nihropenres.14741.r32010

**Published:** 2024-06-27

**Authors:** Daniel Ebbs

**Affiliations:** 1 Yale University, New Haven, Connecticut, USA

Thank you for this opportunity to review your article, *A Multiple criteria decision analysis to establish the use cases and candidate point of care tests to enter into a platform trial of multiple in vitro diagnostic point of care tests in the prehospital environment.* This is an important and timely study. The use of POCTs has the potential to serve as early triage for several medical conditions as discussed in this article. This manuscript aims to design an MCDA to set priority to potential relevant POCTs in the prehospital setting which can guide future stakeholders for implementation.

 Intro: Reliance on in vitro lab for medical decision making is highly over-exaggerated, as referenced by Rohr in “The Value of In Vitro Diagnostics..” in this introduction. This one citation is used as a supporting argument in the introduction, which interviews highly sub-specialized physicians (cardiologist/oncologists) who rely on POCTs for very specific chronic medical conditions. Although medical providers are highly reliant on these diagnostics, 60-70% is extreme, and this is stated without any context.

 Methods: The use of multiple criteria decision analysis (MCDA) is both innovative and appropriate for this study. Appropriate guidelines followed via ISPOR.

 For article review, the gold standard for scoping reviews as outlined by more contemporary sources, such as Joanna Brigg Institute, typically requires two reviewers to review articles for inclusion; having one reviewer select all inclusion can introduce bias which should be noted in discussion. It is noted that a second reviewer was utilized for exclusion review.

 The stakeholder survey methods and results are not representative of experts; the breadth of POCTs in the prehospital setting, particularly among EMS, is novel and new to the last decade, and there is limited data in the form of studies, as you noted (cited in extended file 1) , on most prehospital POCTs. Please expand on how reaching out to random authors who publish on this or applying snowball methods can be synonymous to expertise in the field. Please expand on the limitation section that describes stakeholders.

 Discussion should also discuss *serious* limitations in clinical utility/effectiveness of each POCT and discuss the lack in quality evidence overall in the impact assessment. For example, Troponin, which scored upward of 3 on the evidence of clinical effectiveness, has only three RCTs, and limited overall evidence towards its benefit; yet it is cited among experts that this is above moderate.

Is the work clearly and accurately presented and does it cite the current literature?

Yes

If applicable, is the statistical analysis and its interpretation appropriate?

Partly

Are all the source data underlying the results available to ensure full reproducibility?

Yes

Is the study design appropriate and is the work technically sound?

Yes

Are the conclusions drawn adequately supported by the results?

Partly

Are sufficient details of methods and analysis provided to allow replication by others?

Partly

Reviewer Expertise:

Prehospital emergency services, Prehospital POCTs, Prehospital Predictive Modeling

I confirm that I have read this submission and believe that I have an appropriate level of expertise to confirm that it is of an acceptable scientific standard, however I have significant reservations, as outlined above.

# Reviewer report: Reviewer response for version 1

**DOI:** 10.3310/nihropenres.14741.r31901

**Published:** 2024-06-11

**Authors:** Rudolf T Tolsma

**Affiliations:** 1 Emergency Medical Service, Ambulance IJsselland, Zwolle, The Netherlands

Thank you for the opportunity to review this article.

 The researchers did a very thorough job and have also given an excellent coverage of this in this article. In developing an MDCA, the researchers, with input from Public Contributors and stakeholders, have been able to identify 3 key use cases that could be further explored in a platform trial in the (near) future. The topic focuses on important developments in prehospital care, namely the use of point-of-care diagnostics. The use of these diagnostics helps in the decision-making of healthcare professionals, strives for the right care in the right place for patients and offers opportunities to reduce overcrowding in emergency departments and improve ambulance availability.

Is the work clearly and accurately presented and does it cite the current literature?

Yes

If applicable, is the statistical analysis and its interpretation appropriate?

Not applicable

Are all the source data underlying the results available to ensure full reproducibility?

Yes

Is the study design appropriate and is the work technically sound?

Yes

Are the conclusions drawn adequately supported by the results?

Yes

Are sufficient details of methods and analysis provided to allow replication by others?

Yes

Reviewer Expertise:

Prehospital care, riskstratification, point of care testing in healthcare

I confirm that I have read this submission and believe that I have an appropriate level of expertise to confirm that it is of an acceptable scientific standard.
